# Prevalence and Determinants of Hypertension Awareness, Treatment, and Control in Botswana: A Nationally Representative Population-Based Survey

**DOI:** 10.1155/2020/8082341

**Published:** 2020-05-31

**Authors:** Neo M. Tapela, Lei Clifton, Gontse Tshisimogo, Moagi Gaborone, Tebogo Madidimalo, Virginia Letsatsi, Tiny Masupe, Mosepele Mosepele, Joseph Makhema, Shahin Lockman, David J. Hunter

**Affiliations:** ^1^Nuffield Department of Population Health, University of Oxford, Oxford, UK; ^2^Botswana Harvard AIDS Institute Partnership, Gaborone, Botswana; ^3^National NCD Program, Ministry of Health and Wellness, Gaborone, Botswana; ^4^World Health Organization, Gaborone, Botswana; ^5^University of Botswana, Gaborone, Botswana; ^6^Harvard T.H. Chan School of Public Health, Boston, MA, USA

## Abstract

**Introduction:**

Hypertension is a leading risk factor for cardiovascular mortality and an emerging public health concern in sub-Saharan Africa. Few studies have examined performance on the management of hypertension in this region, where the context may be distinct from other developing regions.

**Objectives:**

We aimed to determine the prevalence and correlates of hypertension, awareness, treatment, and control among adults in Botswana, a middle-income African country undergoing rapid demographic transition and with high HIV burden.

**Methods:**

In this 2014 cross-sectional survey of adults aged 15–69 years, information on sociodemographic characteristics, lifestyle behavior, and medical history was collected through in-person interviews and physical measurements (body mass index and triplicate blood pressure (BP)). Hypertension was defined as self-report of use of antihypertensives in the previous two weeks and/or having elevated BP (≥140/90 mmHg). Multivariable logistic regression was employed to explore factors associated with hypertension, awareness (report of previous diagnosis), treatment (antihypertensives), and control (BP < 140/90).

**Results:**

Our analysis (*N* = 4,007) yielded an age-standardized hypertension prevalence of 30% (95% CI: 28%–32%, *N* = 1,393). Among hypertensives, 54% (50–58%) were unaware of their condition, 45% (40–50%) of those aware were untreated, and 63% (55–70%) of those on medications were suboptimally treated (BP ≥ 140/90 mmHg). A fifth of hypertensives who were diagnosed but not on medications had BP ≥ 180/110 mmHg. Diabetes was the strongest correlate of hypertension and awareness (aOR 4.00, 1.86–8.59; aOR 3.30, 1.44–7.55, respectively). Males were less likely to be aware (aOR 0.62, 0.41–0.94) or controlled (aOR 0.36, 0.16–0.83). Obese individuals were more likely to be treated (aOR 2.17, 1.12–4.22), yet less likely to be controlled (aOR 0.32, 0.15–0.66).

**Conclusions:**

We report the first nationally representative estimates of the hypertension care cascade performance in Botswana, which will support planning and future policy evaluations. Findings contribute to the relatively sparse evidence on this subject and may inform development of innovations that improve quality of hypertension management and adherence support in similar settings.

## 1. Background

Cardiovascular diseases (CVD) are the leading cause of mortality worldwide, with 80% of associated deaths occurring in low- and middle-income countries (LMICs) [1]. Hypertension is the leading risk factor for CVD-related mortality globally contributing to over 7 million premature deaths per year [2]. Addressing hypertension will be instrumental to stemming the burden of CVDs and achieving Sustainable Development Goal target 3.4: to reduce premature mortality due to noncommunicable diseases (NCD) by a third by 2030 [3, 4].

Despite this, and the availability of low-cost treatments for hypertension [5], many individuals with the condition living in LMICs are undiagnosed or suboptimally treated (uncontrolled) [5–7]. Studies have previously described high levels of hypertension control in high income countries (HICs), exceeding 80% among hypertensives on treatment in Canada and the UK [8, 9]. In contrast, these figures are much lower in LMICs [10, 11]. While many studies have described determinants of hypertension [10, 12–14], fewer have explored determinants of hypertension awareness, treatment, and control. This is particularly the case for sub-Saharan Africa, where CVD is the second leading cause of premature mortality [15], prevalence of hypertension among adults ranges from 15% to 42% [10], and the context may be distinct from other LMIC settings [16]. Furthermore, many African studies have used data that were not population-based or not nationally representative [12, 13], and the factors examined have often been limited to sociodemographic and lifestyle behavior.

Given this backdrop, we aimed (a) to determine the prevalence of hypertension awareness, treatment, and control in Botswana, a middle-income African country with a population of 2 million undergoing rapid demographic transition and with high prevalence of HIV (19%), and (b) to identify correlates of these hypertension care cascade categories among adults, including lifestyle advice and comorbidity. Characterizing gaps in the hypertension care cascade and factors associated with uncontrolled hypertension may inform future research and policy approaches in Botswana and other resource-limited settings with emerging hypertension burden.

## 2. Methods

### 2.1. Design and Study Population

We conducted secondary analysis of data from a population-based cross-sectional NCD risk factors survey conducted in Botswana between July and December 2014. The survey followed established World Health Organization (WHO) STEPwise NCD risk factor surveillance (STEPS) methodology [17] and employed multistage cluster sampling design to enroll a nationally representative sample of 15–69-year-old citizens residing in the country. The 2011 Population and Housing Census served as the sampling frame, with a population of 2,024,904, residing in 4,845 enumeration areas. Enumeration areas, which correspond to villages in rural areas and neighborhoods in urban areas, were primary sampling units of which a random sample was selected across the 27 health districts, based on probability proportional to size sampling. We note that the selection is not stratified by district. From these, a simple random sample of households (secondary sampling units) was selected, and subsequently a single age-eligible occupant was randomly selected per household using the Kirsch algorithm contained in a hand-held Android device. A complete analysis weight was computed to account for probability of selection at each sampling stage and nonresponse and the differences in age and sex distributions between the sample and Botswana reference 2011 population.

### 2.2. Data Collection

The survey was implemented by a trained team of 144 Ministry of Health and Wellness (MOH) health providers, supported by the MOH National Program team and WHO officers. Data were entered directly on to hand-held personal digital assistants, and collection involved three steps: (1) in-person interview, (2) anthropometric (height, weight, and waist circumference) and blood pressure (BP) measurements, and (3) collection of a fasting blood specimen for glucose and lipid profile testing. The interview employed a structured questionnaire, gathering information on participant sociodemographic characteristics, lifestyle behavior, hypertension and other medical history, and prior receipt of advice on lifestyle behavior modification. BP was measured using an automated validated [18] device (OmronM2, Omron Healthcare, Japan) and with three adult cuff sizes available. Following an initial rest of 15 minutes, three BP measurements were taken at least 5 minutes apart, with the participant in sitting position.

### 2.3. Definitions

Hypertension was defined as self-report of being on antihypertensive medications in the previous two weeks and/or having elevated BP (mean systolic blood pressure (SBP) ≥ 140 mmHg or mean diastolic blood pressure (DBP) ≥ 90 mmHg) during survey measurement. Three further binary hypertension status categories (care cascade phases) were of interest: awareness, treatment, and control. Awareness was defined as report of ever being previously informed of a hypertension diagnosis by a health professional. Treatment was report as being on antihypertensive medications in the previous two weeks. Control was having measured mean SBP < 140 and mean DBP < 90. These categories produce mutually exclusive groups of uncontrolled hypertensives previously described in other studies [10, 19–21]: unaware, untreated, or suboptimally treated. Hypertension severity was categorized into three stages following thresholds from the Seventh Report of the Joint National Committee on Prevention, Detection, Evaluation, and Treatment of High Blood Pressure [22]: stage I corresponds to SBP 140–159 mmHg or DBP 90–99 mmHg, stage II SBP 160–179 mmHg or DBP 100–110 mmHg, and stage III (also referred to as hypertensive crisis) SBP > 180 mmHg or DBP > 110 mmHg.

Variables available in the STEPS dataset that were included in the analysis were sociodemographic characteristics (age, gender, education, and rural residence), known correlates of raised blood pressure and/or risk factors of CVD (alcohol, smoking, added salt, diet, and obesity), and potential correlates of hypertension control that were less often reported (comorbidities, receipt of lifestyle advice). Known diabetes was defined as self-report of being informed by a health professional of having diabetes or of being on glycemic medications in the previous two weeks. Participants were considered having “other comorbidities” if they reported history of asthma, cancer, renal disease, “depression or other mental illnesses,” or HIV. Specific enquiry was made for the above listed chronic conditions with the exception of HIV, where self-report involved volunteered response to a general question: “do you have any other medical conditions?” (and data entered in free text such as “HIV,” “RVD” (retroviral disease), and “on ART” (antiretroviral therapy)).

BMI was computed using the formula of weight divided by height squared (kg/m^2^). BMI was classified as normal (≤25 kg/m^2^), overweight (25.1–30 kg/m^2^), and obese (>30 kg/m^2^). Current smoking was any use of smoked tobacco in the preceding 30 days. Binge use of alcohol was report of drinking six or more units of alcohol in one occasion during the preceding 30 days. Added salt at meals was a response other than “never” (i.e., selecting “rarely,” “sometimes,” “often,” or “always”) to the question, “how often do you add salt or salty sauce to your food just before or during eating?” Low fruit or vegetable intake was defined as consuming, on average, less than five servings of fruits or vegetables per day. Participants were considered to have received lifestyle advice if they responded yes to any of the options for the question, “during the past three years, has a doctor or any other health worker advised you to do any of the following: quit using tobacco, reduce salt, eat at least five servings of fruit and/or vegetables each day, start or do more physical activity?” Age groups were categorized to reflect varying CVD risk and conventional demographic groups: 15–29 years, 30–49 years, and 50–69 years. Allocation of rural/urban district status was based on Botswana's 2011 National Population and Housing Census.

### 2.4. Statistical Analysis

Descriptive analyses were performed, producing proportions for categorical variables and medians/means for continuous variables. Age- and sex-standardized prevalence estimates for the care cascade phases of hypertension, awareness (among all hypertensives), treatment (among all hypertensives and among those aware), and control (among all hypertensives and among those treated) were computed for the overall population and for subgroups based on predictors of interest. Univariable logistic regression was performed to explore unadjusted relationships between explanatory variables and each hypertension care cascade phase. Multivariable logistic regression models were then fitted to compute fully adjusted odds ratios, with 95% confidence intervals. All analyses were performed using STATA v15 (Stata Corporation, College Station, Texas, USA) and factoring in study design and weights (svyset and svy commands).

### 2.5. Ethics

This study was approved by Botswana MOH's Health Research Development Committee. Written informed consent was required for participation in this study. Participants with elevated BP were referred to their local clinic for further review (urgently for those with BP > 180/110).

## 3. Results

**Figure 1:**
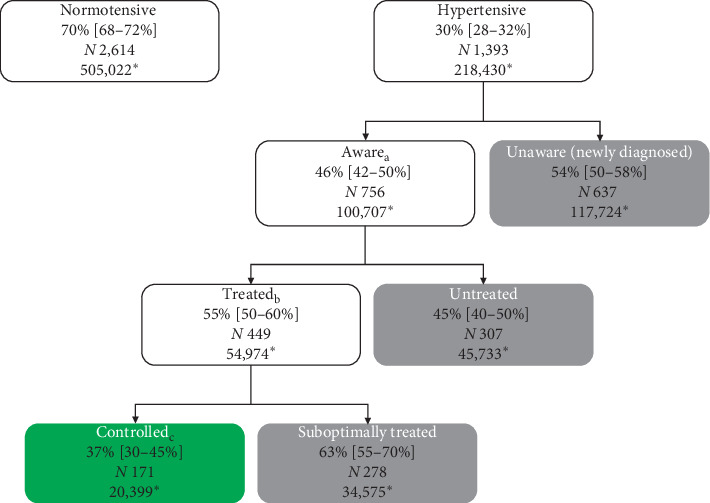
Flow chart for selection of analytic dataset.

**Figure 2:**
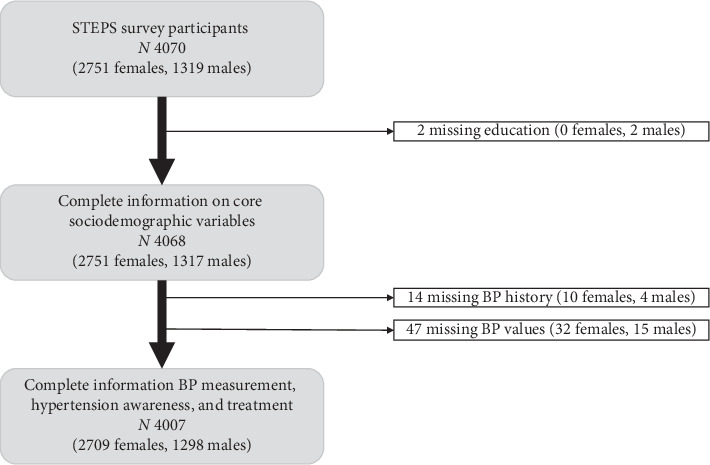
Age-standardized hypertension prevalence, awareness, treatment, and control in the general adult population aged 15–69 years: (a) among participants who are hypertensive; (b) among participants who are aware/diagnosed; (c) among participants who are aware and on medications/treated. Hypertension was defined as self-report of being on antihypertensives in the last two weeks (treated), or having elevated BP based on survey BP measurement (mean SBP ≥ 140 mmHg or mean DBP ≥ 90 mmHg). Awareness was defined as self-report of being previously informed of hypertension diagnosis by a health professional. Control was defined as measured BP < 140/90. ^*∗*^Number of individuals represented in the category based on Botswana's 2011 Population Census.

A total of 4,070 participants (64% of target sample size), spanning 227 (76% of target) enumeration areas, provided consent and were enrolled in the survey (Tables 1 and 2). Analysis was restricted to participants with nonmissing core variables of gender, age, education, rural residence, hypertension history (medication use, whether previously informed of diagnosis), and measured BP-yielding a final analytic dataset of 4,007 participants (Figures 1 and 2).

### 3.1. Participant Characteristics

Participant sociodemographic, behavioral and clinical characteristics are summarized in Tables 3 and 4. Participants were young (median age 34 (25–47) years, 79% aged < 50 years) and predominantly female (2,709, 68%). A third (1,319, 33%) had primary schooling or less, while 82% (3,297) resided in rural areas. With respect to cardiovascular risk factors, 38% (1,524) of participants were overweight or obese, 13% were current smokers, and 3% were known diabetics.

### 3.2. Prevalence of Hypertension, Awareness, Treatment, and Control

**Figure 3:**
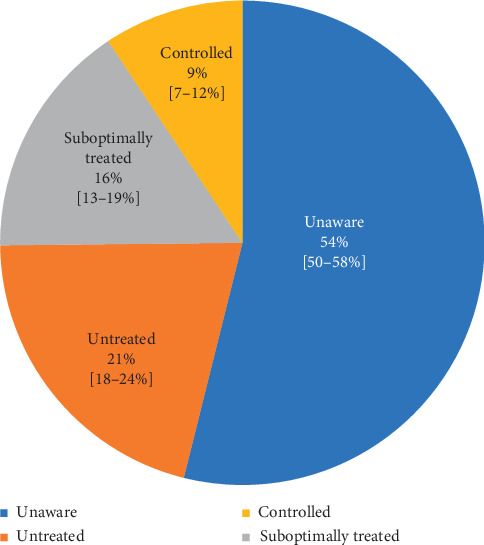
Age-standardized proportions of undiagnosed, untreated, and suboptimally controlled blood pressure among adult hypertensives. The common denominator used here was the number of participants with hypertension, defined as self-report of being on antihypertensives in the last two weeks (treated) or having elevated blood pressure (BP) based on survey measurement (mean SBP ≥ 140 mmHg or mean DBP ≥ 90 mmHg). Hypertensives were divided into mutually exclusive groups. Unaware hypertensives were those who had elevated BP, yet had not previously been informed of a hypertension diagnosis by a health professional. Untreated hypertensives were those who had been previously informed of a hypertension diagnosis but were not on medications in the past 2 weeks and had elevated BP. Suboptimally treated hypertensives were those who had been previously informed of a diagnosis and were on medications, yet had elevated BP. Controlled hypertensives were those who had been previously informed of diagnosis, were on medications and had BP < 140/90.

We found a high burden of hypertension, with an estimated prevalence of 30% (95% CI: 28%–32%, *N* = 1 393) in the general adult population, 58% (53–64%) among those aged 50–69 years, and 77% (66–86%) among known diabetics (Table 1). We also found large drop-offs in the hypertension care cascade (Figure 1). Over half (54%, 50–58%) of hypertensives were unaware of their condition, 45% (40–50%) of those who were aware were not on medications, and 63% (55–70%) of those treated had BP above target of 140/90 (suboptimally treated). Based on Botswana's 2011 population structure, these figures translate to 218,430 prevalent hypertensives in the country, out of whom 117,724 were unaware, 45,733 were untreated, and 34,575 were suboptimally treated (Figure 1). All told, these results indicate that, out of all prevalent hypertensives, only 9% (7–12%) in Botswana are controlled while the remaining 91% (88–92%) are either undiagnosed, untreated, or suboptimally treated (Figure 3).

Table 5 summarizes severity of BP elevation among uncontrolled hypertensives. 8% (5–12%) of unaware hypertensives had hypertensive crisis (stage III, BP > 180/110) and a further 18% (14–23%) had moderately elevated BP (stage II, BP 160–179/100–109). Nearly a fifth of hypertensives who were diagnosed but untreated (19%, 12–28%) and a fifth of those suboptimally treated (19%, 13–28%) had hypertensive crisis.

### 3.3. Factors Associated with Hypertension, Awareness, Treatment and Control

Individuals who were older, less educated, current smokers, obese, or known diabetic were more likely to be hypertensive, based on univariable regression analysis (Table 1). Those who were older, female, or obese were more likely to be aware and to be treated. Having diabetes or other comorbidity was associated with higher likelihood of hypertension awareness. Binge use of alcohol was associated with lower likelihood of awareness and of treatment. Receipt of lifestyle risk factor advice was associated with higher likelihood of awareness and treatment and lower likelihood of hypertension control.

In fully adjusted multivariable logistic regression analysis (Table 2), older age (aOR 1.04, 95% CI: 1.03–1.06), being male (aOR 1.30, 1.03–1.65), obesity (aOR 2.96, 2.16–4.04), overweight (aOR 1.56, 1.20–2.04), and comorbid diabetes (aOR 4.00, 1.86–8.59) were independent predictors of hypertension. Current smokers tended to be hypertensive; however, this association did not reach statistical significance (aOR 1.40, 0.98–1.99). Men were less likely to be aware (aOR 0.62, 0.41–0.94) and to achieve control (aOR 0.36, 0.16–0.83) than women. However, diagnosed men were as likely to receive treatment as women. Being obese was associated with higher likelihood of hypertension awareness (aOR 2.27, 1.40–3.67) and treatment (aOR 2.17, 1.12–4.22), yet lower likelihood of control (aOR 0.32, 0.15–0.66). Comorbid diabetes was the strongest predictor of hypertension awareness (aOR 3.30, 1.44–7.55), with a trend towards higher likelihood of treatment and control although this did not reach statistical significance. Receiving lifestyle risk factor advice in the past three years was strongly associated with higher likelihood of treatment (aOR 4.98, 2.42–10.23) but, paradoxically, lower likelihood of hypertension control (0.27, 0.08–0.83). Binge alcohol users were less likely to be treated (aOR 0.41, 0.18–0.94).

## 4. Discussion

We found a prevalence of hypertension that is comparable to other estimates in the region [10–12, 14, 23, 24]. Diabetes was the strongest predictor of hypertension, with gender, age, and obesity also associated with hypertension. This provides additional support of findings from other studies describing these predictors of hypertension.

Our results indicate a large antihypertensive treatment gap. In Botswana, it is possible to delink access to medical treatment from financial constraints because of the country's favorable context as a middle-income country with free healthcare for citizens and where over 95% of the population resides within 8 km of a health facility [25]. That nearly half of hypertensives who had previously been diagnosed were untreated, and that a fifth of those untreated were in hypertensive crisis (BP > 180/110) is sobering. This contradicts the common misperceptions that hypertensives who are untreated have only mildly elevated BP, amenable to lifestyle modification alone rather than management with antihypetensive drugs. This finding also emphasizes the importance of better control of BP given that hypertensive emergency (BP ≥ 180/110 with associated evidence of new or worsening target organ damage) is associated with a one-year death rate of over 79% and median survival of 10.4 months if left untreated [26].

The observed treatment gap is likely due to a combination of delivery of poor quality care by providers/health system and limited uptake of available services by patients. Poor quality healthcare, which can be defined as delivery of services that are not compliant with evidence-based guidelines, is a global challenge that contributes to cardiovascular disease-related deaths. In their analysis of the 2016 Global Burden of Disease data, Kruk and colleagues found a toll of 2,358,000 cardiovascular deaths (or 84% of cardiovascular deaths amenable to healthcare) caused by poor-quality care [27]. Urgently needed approaches to address this treatment gap include developing national evidence-based guidelines and training providers on them, strengthening supply chain and facility management, and establishing a pathway for follow-up and reassessment of individuals who previously had only mildly elevated BP. In Botswana, the first evidence-based guidelines for management of hypertension at the primary care level were endorsed in 2016, two years following the data here presented. These were based on WHO's Package of Essential NCDs Interventions (WHO PEN) and contained algorithms for BP screening, CV risk assessment (including glucose and lipid profile), lifestyle advice (smoking cessation, diet, and exercise), and risk-based treatment. Treatment algorithms describe use of antihypertensives for BP persistently ≥ 140/90 mmHg (thiazides, +ACE-inhibitors, +calcium channel blockers, and beta-blockers as 1^st^, 2^nd^, 3^rd^, and 4^th^ line respectively) and use of statins and aspirin if 10-year CV risk exceeding 30%. We conducted a survey of 142 government health facilities in early 2017, which revealed that service inputs such as personnel, essential medicines for CV risk management (with the exception of statins), and equipment were generally available at the facility level. Key gaps identified included provider knowledge on hypertension and other chronic NCDs and lack of recall systems for longitudinal follow-up of patients [28].

The authors recognize that five years have elapsed since data collection and that updated assessment of the prevalence of hypertension and its control (and service delivery processes related to control) is due. This could be achieved by incorporating NCDs data elements in other national, population-based surveys that may occur before the next STEPS survey, such as the triennial Botswana AIDS Impact Survey. Our reported analysis would provide a reference baseline against which future analyses and impact evaluation of the 2016 Primary Healthcare guidelines can be compared.

The fact that two-thirds of treated hypertensives had BP ≥ 140/90 (a liberal threshold given evidence that lower BP targets confer cardiovascular benefit), and obese individuals were less likely to achieve control, speaks to the need of couple treatment with strong behavioral supports for medication and healthy lifestyle adherence. The paradoxical association of lifestyle advice with lower likelihood of control in our analysis suggests that this advice, typically delivered as brief mention by providers not trained in motivational counseling, may be a marker of disease that is “difficult” to manage. There is a need to scale up effective approaches for hypertension management adherence support in resource-limited settings. One such approach, not currently available in routine care in Botswana and many developing countries, is home-based BP monitoring and motivational counseling delivered by trained lay health workers supervised by health professionals. This has been demonstrated to be effective in influencing behavior and reducing BP in cluster randomized controlled trials in Argentina [29], Malaysia, and Colombia [30].

With regard to correlates of hypertension care cascade phases, being older, obese, and having diabetes or other comorbidity were all associated with high likelihood of hypertension diagnosis. This is likely due to more interaction with healthcare services of these individuals rendering them more accepting of and available for opportunistic screening. There may also be a contribution of more proactive opportunistic screening by providers who appropriately assess these individuals at higher cardiovascular risk. We also found that hypertensive men were less likely to be aware or controlled than women, a phenomenon described in many other studies [21]. Our findings support the hypothesis that diabetes, as a chronic condition for which regular interaction with healthcare is necessary, provides a gateway for opportunistic screening for hypertension and linkage to treatment. As such it is conceivable that this would be the case for other chronic conditions including HIV, and indeed studies have found higher hypertension awareness [31], treatment [31], and control [32] among HIV-infected individuals. These findings support an approach of integrating hypertension service delivery into existing longitudinal care platforms for HIV and other chronic conditions in order to enhance hypertension detection and management, an approach that has been recommended widely [33, 34].

While our unadjusted analyses found associations between education and hypertension outcomes similarly to some studies [35–37], these associations were not statistically significant in our multivariable analysis. Exploratory analyses did not identify confounding effects of variables not included in some studies: rural residence, comorbidities, and lifestyle advice. In addition to contribution of insufficient power (supported by our 95% confidence Intervals), context factors may be at play. This would be consistent with studies reporting differing relationships between education and hypertension treatment outcomes [38] and a systematic review on the determinants of hypertension noting that the association between education and hypertension is among those inconsistently reported [39].

### 4.1. Limitations

Our study has several notable limitations. The definition of hypertension used in this study excluded individuals who had been informed of hypertension but were not on medications and did not have elevated BP and thus may have underestimated hypertension prevalence. The definition we used was preferred as it yields more conservative estimates and has been used in many other studies [10, 19–21], with which comparisons can be made. As we used the mean for all three BP measurements, there is a possibility that “white coat hypertension” may have falsely raised our estimates for hypertension prevalence. Sensitivity analyses (using only BP values from the 2^nd^ and 3^rd^ measurements) were reassuring in this regard, with findings similar to our primary results. The STEPS survey relied on self-report of antihypertensive treatment which may have resulted in misclassification and underestimation of hypertension prevalence and proportion treated. Only 64% of target sample size was achieved (enrollment was lower in remote, sparsely populated areas with challenging terrain such that survey staff were unable to reach communities), and only a third of the sample was male (males tended to not be home during survey visit times which included early evenings), limiting the sample's representativeness of Botswana's population. Nonetheless, our hypertension prevalence estimates were comparable to those from other studies in the region. The survey, whose primary objective was to estimate prevalence of NCD risk factors, did not include data on some factors known to be associated with hypertension control such as medication adherence, provider training, care facility type, and healthcare utilization. The sample size of treated hypertensives was small such that analysis of correlates of control was insufficiently powered. That said, we were able to examine sociodemographic, behavioral, and clinical factors and document associations that may influence further research and practice.

## 5. Conclusions

We report the first nationally representative estimates of hypertension care cascade in Botswana, which will inform planning and serve as a baseline for future evaluations of national policy. Furthermore, we contribute to the relatively sparse evidence in the African setting on determinants of hypertension control performance, examining less frequently reported correlates. We found that nearly a third of the adult population was hypertensive and that there were large gaps in each care cascade step such that only 9% of all hypertensives were controlled, and a fifth of hypertensives who were diagnosed but untreated were in hypertensive crisis. Male and obese hypertensives were less likely to be controlled, while comorbidities such as diabetes may serve as a gateway to enhance hypertension awareness and treatment. These results highlight the need to accelerate hypertension control efforts in similar settings and may inform development of innovations that improve quality and effectiveness of care and ultimately reduce CVD-related deaths.

## Figures and Tables

**Table 1:** Sociodemographic characteristics of survey participants, by gender (unweighted).

Participant characteristic	All 4,007		Male		Female	
	*N*	%	*N*	%	*N*	%
*Gender*						
Female	2,709	67.6				
Male	1,298	32.4				
Missing	0	0.0				

*Age group*						
15–29 years	1,537	38.4	554	42.7	983	36.3
30–49 years	1,640	40.9	509	39.2	1,131	41.8
50–69 years	830	20.7	235	18.1	595	22.0
Missing	0	0.0	0	0.0	0	0.0
Median age in year (IQR)	34 (25–47)		32 (24–45)		35 (26–47)	

*Marital status*						
Never married	2,600	64.9	874	67.3	1,726	63.7
Married or cohabiting	1,171	29.2	381	29.4	790	29.2
Separated, widowed, or divorced	231	5.8	42	3.2	189	7.0
Missing	5	0.1	1	0.1	4	0.2

*Ethnic group*						
Motswana	3,861	96.4	1,241	95.6	2,620	96.7
Other African	134	3.3	52	4.0	82	3.0
European	2	0.1	0	0.0	2	0.1
Asian	3	0.1	2	0.2	1	0.0
Other	6	0.2	2	0.2	4	0.2
Missing	1	0.0	1	0.1	0	0.0

*Education*						
No formal schooling	373	9.3	126	9.7	247	9.1
Primary (1–7 years)	946	23.6	265	20.4	681	25.1
Secondary (8–12 years)	1,985	49.5	624	48.1	1,361	50.2
Tertiary and higher (>12 years)	703	17.5	283	21.8	420	15.5
Missing	0	0.0	0	0.0	0	0.0

*Employment status*						
Employed	1,086	27.1	465	35.8	621	22.9
Self-employed	483	12.1	226	17.4	257	9.5
Unpaid homemaker	603	15.1	103	7.9	500	18.5
Student or retired	513	12.8	195	15.0	318	11.7
Unemployed	1,320	32.9	309	23.8	1,011	37.3
Missing	2	0.1	0	0.0	2	0.1

*Rural district*						
Urban	710	17.7	236	18.2	474	17.5
Rural	3,297	82.3	1,062	81.8	2,235	82.5
Missing	0	0.0	0	0.0	0	0.0

**Table 2:** Clinical and behavioral characteristics of survey participants, by gender (unweighted).

Participant characteristic	All (4,007)		Male (*N *=* *1,298)		Female (*N = *2,709)	
	*N*	%	*N*	%	*N*	%
*Body mass index*						
Underweight, <18.5	493	12.3	252	19.4	241	8.9
Nl, 18.5 ≤ 25	1,881	46.9	749	57.7	1,132	41.8
Overweight, 25 ≤ 30	866	21.6	216	16.6	650	24.0
Obese, >30	658	16.4	79	6.1	579	21.4
Missing	109	2.7	2	0.2	107	4.0

*Known diabetes*						
Not diabetic	3,898	97.3	1,268	97.7	2,630	97.1
Diabetic	109	2.7	30	2.3	79	2.9
Missing	0	0.0	0	0.0	0	0.0

*Other comorbidities (asthma, cancer, renal disease, “depression or other mental illnesses,” or HIV)*						
No	3,404	85.0	1,147	88.4	2,257	83.3
Yes	602	15.0	151	11.6	451	16.7
Missing	1	0.0	0	0.0	1	0.0

*Smoking status*						
Nonsmoker	3,491	87.1	892	68.7	2,599	95.9
Current smoker	516	12.9	406	31.3	110	4.1
Missing	0	0.0			0	0.0

*Binge alcohol use*						
No	3,459	86.3	946	72.9	2,513	92.8
Yes	548	13.7	352	27.1	196	7.2
Missing	0	0.0	0	0.0	0	0.0

*Low fruit/vegetable intake*						
No	171	4.3	42	3.2	129	4.8
Yes	3,695	92.2	1,202	92.6	2,493	92.0
Missing	141	3.5	54	4.2	87	3.2

*Adding table salt*						
No	2,179	54.4	666	51.3	1,513	55.9
Yes	1,820	45.4	630	48.5	1,190	43.9
Missing	8	0.2	2	0.2	6	0.2

*Lifestyle advice*						
No	1,167	29.1	484	37.3	683	25.2
Yes	2,840	70.9	814	62.7	2026	74.8
Missing	0	0.0	0	0.0	0	0.0

Binge use of alcohol was defined as drinking six or more units of alcohol in one occasion during the preceding 30 days. Added salt at meals was defined as a response other than “never” in response to the question, “how often do you add salt or salty sauce to your food just before or during eating?”. Other comorbidities: self-reported history asthma, cancer, renal disease, “depression or other mental illnesses,” or HIV. The fully adjusted model included all variables listed above but for “diagnosis in previous 12 months” for hypertensives not previously diagnosed. Participants were considered to have received lifestyle advice if they responded yes to any of the options for the question, “during the past three years, has a doctor or any other health worker advised you to do any of the following, quit using tobacco, reduce salt, eat at least five servings of fruit and/or vegetables each day, start or do more physical activity?”

**Table 3:** Distribution of severity of blood pressure elevation among uncontrolled hypertensives (those who are unaware, untreated, and suboptimally treated).

BP measurement	Unaware (*N *=* *637)				Untreated (*N *=* *307)				Suboptimally treated (*N *=* *278)			
	%	95%	CI	*N*	%	95%	CI	*N*	%	95%	CI	*N*
Stage1 BP (SBP 140–159)	0.744	0.69	0.791	446	0.56	0.471	0.645	168	0.529	0.444	0.6119	140
Stage2 BP (SBP 160–179)	0.181	0.144	0.225	139	0.253	0.193	0.325	86	0.277	0.214	0.3503	89
Stage3 BP (SBP ≥ 180)	0.075	0.047	0.119	52	0.187	0.121	0.279	53	0.194	0.131	0.2784	49

Unaware hypertensives were those who had elevated BP yet had not previously been informed of a hypertension diagnosis by a health professional. Untreated hypertensives were those who had been previously informed of a hypertension diagnosis but were not on medications in the past 2 weeks and had elevated BP. Suboptimally treated hypertensives were those who had been previously informed of a diagnosis and were on medications yet had elevated BP.

**Table 4:** Prevalence and univariable logistic regression analysis of factors associated with hypertension, awareness, treatment, and control among adults in Botswana.

	Hypertension (*n* = 1,393)						Awareness, among hypertensives (*n* = 756)^a^					
	*N*	%	OR	95%	CI	*p*	*N*	%	OR	95%	CI	*p*
*Gender*	**4007**						**1393**					
Female (ref)	2709	29.0%					963	56.6%				
Male	1298	31.3%	1.12	0.93	1.34		430	36.7%	0.44	0.32	0.62	^*∗∗∗*^

*Age group*	**4007**						**1393**					
15–29 years (ref)	1537	19.3%					266	24.8%				
30–49 years	1640	34.9%	2.24	1.76	2.85	^*∗∗∗*^	589	49.9%	3.02	1.89	4.81	^*∗∗∗*^
50–69 years	830	58.4%	5.88	4.36	7.93	^*∗∗∗*^	538	66.2%	5.95	3.80	9.30	^*∗∗∗*^

*Highest education*	**4007**						**1393**					
None (ref)	373	43.6%					198	60.2%				
Primary	946	42.2%	0.95	0.64	1.40		465	47.3%	0.59	0.36	0.98	^*∗*^
Secondary	1985	24.5%	0.42	0.30	0.59	^*∗∗∗*^	521	40.4%	0.45	0.27	0.73	^*∗∗*^
Tertiary or higher	703	27.8%	0.50	0.35	0.72	^*∗∗∗*^	209	50.4%	0.67	0.38	1.18	

*Residence type*	**4007**						**1393**					
Urban (ref)	710	27.7%					231	49.8%				
Rural	3297	31.1%	1.17	0.91	1.51		1162	45.0%	0.83	0.55	1.24	

*Current smoking*	**4007**						**1393**					
No (ref)	3491	28.3%					1201	48.8%				
Yes	516	38.6%	1.59	1.18	2.16	^*∗∗*^	192	37.4%	0.63	0.38	1.04	

*Binge alcohol use*	**4007**						**1393**					
No (ref)	3459	29.2%					1203	48.4%				
Yes	548	34.5%	1.28	0.93	1.74		190	37.6%	0.64	0.41	1.00	^*∗*^

*Adding table salt*	***3999***						***1391***					
No (ref)	2179	33.7%					838	51.7%				
Yes	1820	26.6%	0.71	0.58	0.87		553	38.5%	0.58	0.41	0.83	^*∗∗*^

*Body mass index*	***3898***						***1376***					
Normal, BM I< 25 (ref)	2374	24.4%					618	33.1%				
Overweight, BMI 25–30	866	38.6%	1.95	1.53	2.49	^*∗∗∗*^	379	58.3%	2.83	1.87	4.29	^*∗∗∗*^
Obese, BMI > 30	658	54.3%	3.68	2.82	4.80	^*∗∗∗*^	379	65.9%	3.91	2.65	5.75	^*∗∗∗*^

*Known diabetes*	**4007**						**1393**					
No (ref)	3898	29.2%					1310	44.0%				
Yes	109	77.1%	8.14	4.57	14.52	^*∗∗∗*^	83	84.3%	6.81	2.72	17.05	^*∗∗∗*^

*Other comorbidities*	**4006**						***1392***					
No (ref)	3404	29.6%					1168	43.4%				
Yes	602	33.7%	1.21	0.89	1.64		224	61.0%	2.04	1.34	3.11	^*∗∗*^

*Lifestyle advice*	**4007**						**1393**					
No (ref)	1167	27.2%					331	20.4%				
Yes	2840	31.6%	1.23	0.98	1.55		1062	56.3%	5.01	3.37	7.46	^*∗∗∗*^

	Treatment, among aware hypertensives (*n* = 449)^b^						Control, among treated hypertensives (*n* = 171)^c^					
	*N*	%	OR	95%	CI	*p*	*N*	%	OR	95%	CI	*p*

*Gender*	**756**						**449**					
Female (ref)	585	61.2%					363	40.6%				
Male	171	45.4%	0.53	0.33	0.84	^*∗*^	86	30.7%	0.65	0.30	1.39	

*Age group*	**756**						**449**					
15–29 years (ref)	80	28.5%					23	48.4%				
30–49 years	310	50.3%	2.53	1.18	5.44	^*∗*^	155	36.1%	0.60	0.18	1.98	
50–69 years	366	71.0%	6.14	2.88	13.09	^*∗∗∗*^	271	35.8%	0.60	0.21	1.73	

*Highest education*	**756**						**449**					
None (ref)	124	64.4%					85	27.1%				
Primary	284	62.5%	0.92	0.54	1.56		183	37.3%	1.60	0.80	3.19	
Secondary	239	46.9%	0.49	0.26	0.93	^*∗*^	117	41.4%	1.90	0.82	4.43	
Tertiary or higher	109	50.5%	0.56	0.28	1.12		64	37.4%	1.61	0.60	4.28	

*Residence type*	**756**						**449**					
Urban (ref)	132	51.2%					77	46.5%				
Rural	624	55.7%	1.20	0.70	2.06		372	34.1%	0.60	0.28	1.26	

*Current smoking*	**756**						**449**					
No (ref)	682	56.8%					415	37.9%				
Yes	74	45.1%	0.62	0.30	1.28		34	33.1%	0.81	0.26	2.53	

*Binge alcohol use*	**756**						**449**					
No (ref)	680	60.0%					420	37.6%				
Yes	76	28.3%	0.26	0.13	0.54	^*∗∗∗*^	29	32.2%	0.79	0.24	2.60	

*Adding table salt*	***755***						***448***					
No (ref)	483	56.5%					295	33.7%				
Yes	272	51.1%	0.81	0.52	1.24		153	43.7%	1.53	0.85	2.74	

*Body mass index*	***745***						***440***					
Normal, BMI < 25 (ref)	267	44.7%					143	46.2%				
Overweight, BMI 25–30	229	53.6%	1.43	0.84	2.43		127	33.8%	0.59	0.27	1.30	
Obese, BMI > 30	249	66.8%	2.48	1.36	4.53	^*∗∗*^	170	28.0%	0.45	0.21	0.96	^*∗*^

*Known diabetes*	**756**						**449**					
No (ref)	685	53.0%					391	35.9%				
Yes	71	69.4%	2.01	0.86	4.68		58	46.2%	1.54	0.61	3.85	

*Other comorbidities*	**756**						**449**	^*∗∗∗*^				
No (ref)	621	54.4%					368	35.5%				
Yes	135	55.2%	1.03	0.57	1.85		81	43.2%	1.38	0.73	2.62	

*Lifestyle advice*	**756**						**449**					
No (ref)	95	23.5%					30	59.2%				
Yes	661	59.1%	4.71	2.26	9.81	^*∗∗∗*^	419	35.8%	0.39	0.14	1.07	

^*∗*^ *p* < 0.05, ^*∗∗*^*p* < 0.001, and ^*∗∗∗*^*p* < 0.0001. Subtotals with missing values are indicated in italics. Binge use of alcohol was defined as drinking six or more units of alcohol in one occasion during the preceding 30 days. Added salt at meals was defined as a response other than “never” in response to the question, “how often do you add salt or salty sauce to your food just before or during eating?” Other comorbidities: self-reported history asthma, cancer, renal disease, “depression or other mental illnesses,” or HIV. Participants were considered to have received lifestyle advice if they responded yes to any of the options for the question, “during the past three years, has a doctor or any other health worker advised you to do any of the following, quit using tobacco, reduce salt, eat at least five servings of fruit and/or vegetables each day, start or do more physical activity?”

**Table 5:** Multivariable logistic regression of factors associated with hypertension, awareness, treatment, and control among adults in Botswana.

	Hypertension (*n* = 1,393)				Awareness, among hypertensives (*n* = 756)				Treatment, among aware hypertensives (*n* = 449)				Control, among treated hypertensives (*n* = 171)			
	aOR	95%	CI	*p*	aOR	95%	CI	*p*	aOR	95%	CI	*p*	aOR	95%	CI	*p*
Male	1.30	1.03	1.65	^*∗*^	0.62	0.41	0.94	^*∗*^	0.91	0.50	1.69		0.36	0.16	0.83	^*∗*^
Age	1.04	1.03	1.06	^*∗∗∗*^	1.05	1.03	1.06	^*∗∗∗*^	1.07	1.04	1.09	^*∗∗∗*^	1.00	0.97	1.03	
Secondary school or higher	0.86	0.66	1.12		1.46	0.95	2.26		1.49	0.80	2.77		1.34	0.56	3.23	
Rural residence	1.09	0.84	1.40		0.99	0.64	1.54		1.07	0.61	1.88		0.74	0.36	1.51	
Current smoking	1.40	0.98	1.99		0.93	0.5	1.73		1.65	0.69	3.91		1.75	0.56	5.44	
Binge alcohol use	1.21	0.8	1.81		1.28	0.73	2.25		0.41	0.18	0.94	^*∗*^	0.99	0.22	4.41	
Adding table salt	0.80	0.63	1.01		0.71	0.50	1.00		0.95	0.61	1.49		1.78	1.01	3.16	
Low fruit/vegetable intake	0.77	0.44	1.38		0.99	0.38	2.53		1.24	0.48	3.20		0.47	0.15	1.50	

BMI < 25 (ref)																
BMI 25–30	1.56	1.20	2.04	^*∗∗*^	1.66	1.05	2.65	^*∗*^	0.94	0.52	1.68		0.70	0.32	1.56	
BMI > 30	2.96	2.16	4.04	^*∗∗∗*^	2.27	1.40	3.67	^*∗∗*^	2.17	1.12	4.22	^*∗*^	0.32	0.15	0.66	^*∗∗*^

Known diabetes	4.00	1.86	8.59	^*∗∗∗*^	3.30	1.44	7.55	^*∗∗*^	1.62	0.74	3.56		1.96	0.81	4.74	
Other comorbidities	0.93	0.66	1.31		1.85	1.06	3.23	^*∗*^	1.15	0.67	1.97		1.59	0.87	2.89	
Lifestyle advice	N/A	N/A	N/A		N/A	N/A	N/A		4.98	2.42	10.2	^*∗∗∗*^	0.27	0.08	0.83	^*∗*^

^*∗*^ *p* < 0.05, ^*∗∗*^*p* < 0.001, and ^*∗∗∗*^*p* < 0.0001. BMI = body mass index. Binge use of alcohol was defined as drinking six or more units of alcohol in one occasion during the preceding 30 days. Added salt at meals was defined as a response other than “never” in response to the question, “how often do you add salt or salty sauce to your food just before or during eating?” Other comorbidities: self-reported history asthma, cancer, renal disease, “depression or other mental illnesses,” or HIV. Participants were considered to have received lifestyle advice if they responded yes to any of the options for the question, “during the past three years, has a doctor or any other health worker advised you to do any of the following, quit using tobacco, reduce salt, eat at least five servings of fruit and/or vegetables each day, start or do more physical activity?” The fully adjusted models included all variables listed in the table for a given hypertension status outcome.

## Data Availability

The data used to support the findings of this study were from the Botswana National NCD Survey conducted in 2014. Requests for survey data can be made to the Botswana Ministry of Health and Wellness.

## References

[1] WHO (2014). *WHO Global Status Report on NCDs 2014*.

[2] Lawes C. M. M., Hoorn S. V., Rodgers A., Society I. (2008). Global burden of blood-pressure-related disease, 2001. *The Lancet*.

[3] Kruk M. E., Gage A. D., Arsenault C. (2018). High-quality health systems in the Sustainable Development Goals era: time for a revolution. *The Lancet Global Health*.

[4] Bennett J. E., Stevens G. A., Mathers C. D. (2018). NCD Countdown 2030: worldwide trends in non-communicable disease mortality and progress towards Sustainable Development Goal target 3.4. *The Lancet*.

[5] Ettehad D., Emdin C. A., Kiran A. (2016). Blood pressure lowering for prevention of cardiovascular disease and death: a systematic review and meta-analysis. *The Lancet*.

[6] Chow C. K. (2013). Prevalence, awareness, treatment, and control of hypertension in rural and urban communities in high-, middle-, and low-income countries. *JAMA*.

[7] Wirtz V. J., Kaplan W. A., Kwan G. F., Laing R. O. (2016). Access to medications for cardiovascular diseases in low- and middle-income countries. *Circulation*.

[8] Hyman D. J., Pavlik V. N. (2001). Characteristics of patients with uncontrolled hypertension in the United States. *New England Journal of Medicine*.

[9] Sarganas G., Neuhauser H. K. (2016). Untreated, uncontrolled, and apparent resistant hypertension: results of the German health examination survey 2008-2011. *The Journal of Clinical Hypertension*.

[10] Ataklte F., Erqou S., Kaptoge S., Taye B., Echouffo-Tcheugui J. B., Kengne A. P. (2015). Burden of undiagnosed hypertension in sub-saharan Africa. *Hypertension*.

[11] Beaney T., Schutte A. E., Tomaszewski M. (2018). May Measurement Month 2017: an analysis of blood pressure screening results worldwide. *The Lancet Global Health*.

[12] Musinguzi G., Nuwaha F. (2013). Prevalence, awareness and control of hypertension in Uganda. *PLoS One*.

[13] Dzudie A., Kengne A. P., Muna W. F. T. (2012). Prevalence, awareness, treatment and control of hypertension in a self-selected sub-Saharan African urban population: a cross-sectional study. *BMJ Open*.

[14] Damasceno A., Azevedo A., Silva-Matos C., Prista A., Diogo D., Lunet N. (2009). Hypertension prevalence, awareness, treatment, and control in Mozambique. *Hypertension*.

[15] Mayosi B. M., Flisher A. J., Lalloo U. G., Sitas F., Tollman S. M., Bradshaw D. (2009). The burden of non-communicable diseases in South Africa. *The Lancet*.

[16] Kruk M. E., Nigenda G., Knaul F. M. (2015). Redesigning primary care to tackle the global epidemic of noncommunicable disease. *American Journal of Public Health*.

[17] World Health Organization (2017). *The WHO STEPwise Approach to Noncommunicable Diseases Risk Factor Surveillance*.

[18] Asmar R., Khabouth J., Topouchian J., El Feghali R., Mattar J. (2010). Validation of three automatic devices for self-measurement of blood pressure according to the international protocol: the Omron M3 intellisense (HEM-7051-E), the Omron M2 compact (HEM 7102-E), and the Omron R3-I plus (HEM 6022-E). *Blood Pressure Monitoring*.

[19] Berry K. M., Parker W.-a., Mchiza Z. J. (2017). Quantifying unmet need for hypertension care in South Africa through a care cascade: evidence from the SANHANES, 2011-2012. *BMJ Global Health*.

[20] Wozniak G., Khan T., Gillespie C. (2016). Hypertension control cascade: a framework to improve hypertension awareness, treatment, and control. *The Journal of Clinical Hypertension*.

[21] Geldsetzer P., Manne-Goehler J., Marcus M.-E. (2019). The state of hypertension care in 44 low-income and middle-income countries: a cross-sectional study of nationally representative individual-level data from 1·1 million adults. *The Lancet*.

[22] Chobanian A. V., Bakris G. L., Black H. R. (2003). The Seventh report of the Joint national committee on prevention, detection, evaluation, and treatment of high blood pressure. *JAMA*.

[23] Sarfo F. S., Mobula L. M., Burnham G. (2018). Factors associated with uncontrolled blood pressure among Ghanaians : evidence from a multicenter hospital-based study. *PLoS One*.

[24] Mohamed S. F., Mutua M. K., Wamai R. (2018). Prevalence, awareness, treatment and control of hypertension and their determinants: results from a national survey in Kenya. *BMC Public Health*.

[25] Ministry of Health (2010). *The Essential Health Service Package for Botswana*.

[26] Whelton P. K., Carey R. M., Aronow W. S. (2018). Response to letter to editor “2017 ACC/AHA/AAPA/ABC/ACPM/AGS/APhA/ASH/ASPC/NMA/PCNA guideline for the prevention, detection, evaluation, and management of high blood pressure in adults”. *Journal of the American Society of Hypertension*.

[27] Kruk M. E., Gage A. D., Joseph N. T., Danaei G., García-Saisó S., Salomon J. A. (2018). Mortality due to low-quality health systems in the universal health coverage era: a systematic analysis of amenable deaths in 137 countries. *The Lancet*.

[28] Tapela N. M., Tshisimogo G., Shatera B. P. (2019). Integrating noncommunicable disease services into primary health care, Botswana. *Bulletin of the World Health Organization*.

[29] He J., Irazola V., Mills K. T. (2017). Effect of a community health worker–led multicomponent intervention on blood pressure control in low-income patients in Argentina: a randomized clinical trial. *JAMA*.

[30] Schwalm J.-D., McCready T., Lopez-Jaramillo P. (2019). A community-based comprehensive intervention to reduce cardiovascular risk in hypertension (HOPE 4): a cluster-randomised controlled trial. *The Lancet*.

[31] Manne-goehler J., Siedner M. J., Montana L. (2019). Hypertension and diabetes control along the hiv care cascade in rural South Africa. *Journal of the International AIDS Society*.

[32] Kwarisiima D., Atukunda M., Owaraganise A. (2019). Hypertension control in integrated hiv and chronic disease clinics in Uganda in the search study. *BMC Public Health*.

[33] Haldane V., Legido-Quigley H., Chuah F. L. H. (2018). Integrating cardiovascular diseases, hypertension, and diabetes with HIV services: a systematic review. *AIDS Care*.

[34] Rabkin M., Palma A., McNairy M. L. (2018). Integrating cardiovascular disease risk factor screening into HIV services in Swaziland. *AIDS*.

[35] Ware L. J., Chidumwa G., Charlton K., Schutte A. E., Kowal P. (2019). Predictors of hypertension awareness, treatment and control in South Africa: results from the WHO-SAGE population survey (Wave 2). *Journal of Human Hypertension*.

[36] Yang L., Yan J., Tang X., Xu X., Yu W., Wu H. (2016). Prevalence, awareness, treatment, control and risk factors associated with hypertension among adults in southern China, 2013. *PLoS One*.

[37] Chiara T. D., Scaglione A., Corrao S., Argano C., Pinto A., Scaglione R. (2015). Association between low education and higher global cardiovascular risk. *The Journal of Clinical Hypertension*.

[38] Lloyd-sherlock P., Beard J., Minicuci N., Ebrahim S., Chatterji S. (2014). Hypertension among older adults in low- and middle-income countries : prevalence , awareness and control. *International Journal of Epidemiology*.

[39] Bosu W. K., Moses J., Aheto K., Zucchelli E., Reilly S. T. (2019). Determinants of systemic hypertension in older adults in Africa : a systematic review. *BMC Cardiovascular Disorders*.

